# Digital binaural beat music for perceived stress relief among discipline inspection and supervision cadres: a mixed-methods study

**DOI:** 10.3389/fnhum.2026.1892159

**Published:** 2026-08-14

**Authors:** Wen Li, Suli Li, Guoqing Wang

**Affiliations:** 1 School of Teacher Education, Aba Teachers College, Sichuan, China; 2 School of the Arts, Universiti Sains Malaysia, Penang, Malaysia

**Keywords:** binaural beat music, music listening experience, psychological stress, public-sector employees, self-efficacy, work engagement

## Abstract

**Background:**

Discipline inspection and supervision cadres are exposed to high levels of occupational stress, yet convenient and accessible forms of psychological support for this group remain limited.

**Objective:**

This study aimed to examine the associations among self-efficacy, work engagement, subjective music listening experience, and psychological stress among discipline inspection and supervision cadres. It further explored whether subjective music listening experience played a potential mediating role in the relationship between self-efficacy/work engagement and psychological stress.

**Methods:**

A mixed-methods, cross-sectional design was adopted. Using purposive sampling, in-service discipline inspection and supervision cadres from a region of Sichuan Province, China, were recruited. Participants completed measures of psychological stress, self-efficacy, work engagement, and music listening experience after a single session of binaural beat music listening. Quantitative data were analyzed using hierarchical regression and mediation analyses, while qualitative data were examined through thematic analysis.

**Results:**

Self-efficacy/work engagement and music listening experience were both significantly and negatively associated with psychological stress. Moreover, music listening experience explained psychological stress beyond the effects of self-efficacy/work engagement. However, music listening experience did not significantly mediate the relationship between self-efficacy/work engagement and psychological stress. This finding suggests that self-efficacy/work engagement and music listening experience may be associated with psychological stress through relatively independent pathways.

**Significance:**

This study extends research on binaural beat music listening to a high-stress public-sector occupational group and provides preliminary evidence on the role of subjective music listening experience in relation to psychological stress.

## 1 Introduction

Discipline inspection and supervision cadres constitute a high-responsibility occupational group within the Chinese public sector. Their work often involves ethical supervision, case-related tasks, complaint handling, and confidential decision-making, which may expose them to sustained psychological pressure (Zhang et al., 2023; Zhu et al., 2025). Previous studies have shown that this occupational group may experience anxiety, fatigue, and emotional imbalance, which can negatively affect work performance (Song et al., 2014). During the COVID-19 pandemic, nearly half of Chinese civil servants reported symptoms of anxiety (49.24%) or depression (47.1%) (He et al., 2023; Blom et al., 2025). Without appropriate psychological intervention, occupational stress may weaken confidence, reduce work efficiency, and hinder career development. Therefore, identifying effective and accessible stress-reduction strategies has become an urgent task in strengthening the cadre workforce (Lei, 2023; Otu and Sefotho, 2024).

Music therapy has been widely applied in health and well-being contexts because of its accessibility, relatively low cost, and potential benefits for emotional regulation and stress management (Xu, 2020). A substantial body of evidence has shown that music therapy can effectively reduce psychological stress and improve emotional states (Carolan and de Visser, 2018; Nyarubeli et al., 2025; Raittila et al., 2025). For example, a systematic review reported medium to large effects of music therapy on stress-related outcomes, with an effect size of approximately *d* = 0.72 (De Witte et al., 2020, 2022). The World Health Organization has also emphasized the role of music as an emotional regulation resource that can help alleviate physical and psychological suffering (World Health Organization, 2019).

In recent years, binaural beat (BB) music therapy has received increasing scholarly attention. BB are generated by presenting two pure tones with slightly different frequencies separately to each ear, leading the brain to perceive a third, illusory beat frequency. This auditory phenomenon may entrain brainwave activity toward specific frequency ranges and thereby induce relaxation, stress reduction, and other physiological and psychological responses (Pang et al., 2024). Existing studies have suggested that BB therapy may contribute to stress relief, attention enhancement, and sleep improvement (Elnazer, 2026), and may also reduce symptoms of anxiety and depression (Zheng et al., 2023). With the rapid development of artificial intelligence and mobile technologies, smartphone-based BB applications have further expanded the accessibility of emotion regulation tools.

However, research on psychological intervention pathways and outcome evaluation within the discipline inspection and supervision system remains limited (Chen and Xiao, 2020; Wu et al. 2026). Although BB therapy has demonstrated potential for anxiety reduction, its effectiveness has not yet been systematically examined among discipline inspection and supervision cadres, who represent a high-stress occupational group. Therefore, exploring feasible and accessible forms of psychological support for discipline inspection and supervision cadres has become an important and timely research direction.

The significance of this study lies in extending research on BB music listening to discipline inspection and supervision cadres, a high-stress occupational group that has received limited attention in previous studies. The findings may help fill an existing research gap, provide preliminary evidence on the relationship between music listening experience and psychological stress, and offer practical insights into the potential use of digital, non-pharmacological approaches as convenient psychological support for public employees.

## 2 Literature review

### 2.1 Psychological stress among discipline inspection and supervision cadres

Discipline inspection and supervision cadres work in high-intensity and high-risk environments, and their mental health has received increasing attention. From the perspective of occupational characteristics, these cadres undertake important responsibilities such as disciplinary review, investigation, and anti-corruption governance (Chen and Xiao, 2020). Their work involves substantial responsibility, strict procedures, and high confidentiality requirements, which may lead to considerable work stress and psychological burden (He et al., 2023). A case study of human resource management in grassroots discipline inspection and supervision agencies further found that heavy workloads, high psychological stress, and limited mental health service resources were the main challenges faced by this group (Lei, 2023).

Existing studies have also shown that discipline inspection cadres and related public-sector employees are exposed to multiple sources of psychological pressure. University discipline inspection cadres have been found to experience high levels of psychological stress, while effective emotion regulation strategies may help reduce the negative impact of stress on mental health (Zhang et al., 2023). Research on civil servants suggests that their sources of stress are complex, and work-related social pressure, particularly occupational socializing, has been associated with higher levels of job burnout and lower life satisfaction (Song et al., 2014). Psychological resilience, defined as the ability to remain focused and emotionally stable under pressure, has also been identified as an important factor in reducing job burnout among grassroots civil servants (Chen and Xiao, 2020). Overall, existing studies indicate that discipline inspection and supervision cadres face clear mental health risks, highlighting the need for targeted and practically feasible forms of psychological support for this occupational group.

### 2.2 Application of music therapy in stress reduction

Among various psychological support approaches, music therapy is one of the most accessible forms of art therapy because of its ease of use, low cost, and notable effects (Li et al., 2025; Xu, 2020). Previous research has suggested that music may operate through three main pathways: attention diversion, emotion evocation, and physiological response regulation (Xu, 2020). A narrative review focusing on working-age populations found that music therapy may have positive effects on reducing depression, anxiety, and job burnout, and that these effects are not limited to clinically diagnosed high-stress individuals (Raittila et al., 2025). This finding supports the extension of music-based approaches to occupational populations.

In workplace settings, music-based activities have also shown potential for improving employees’ emotional states and reducing stress. A scoping review of 25 workplace music intervention studies found that approaches such as mindful music listening and recreational music activities may help improve employee emotions, reduce anxiety, and lower stress levels (Nyarubeli et al., 2025). In addition, digital forms of psychological support are increasingly accepted among occupational populations because of their convenience, accessibility, and privacy-related advantages (Carolan and de Visser, 2018). These findings suggest that digital music-based approaches may be particularly suitable for high-pressure occupational groups who require flexible and low-burden forms of psychological support.

### 2.3 Binaural beat music, stress regulation, and self-efficacy

BB are produced by presenting pure tones with slightly different frequencies to each ear (Pang et al., 2024). The brain then perceives a third virtual beat frequency, which is considered a potential method for guiding brainwave activity toward specific frequencies (McConnell et al., 2014). In recent years, BB music therapy has gradually gained research interest in the field of psychological intervention.

A systematic review of music and BB interventions for young adults showed that BB produced significant or moderate effects in reducing anxiety and improving emotional states (Elnazer, 2026). Clinical evidence has also indicated that BB music therapy can significantly reduce anxiety and depression among stroke patients with hemiplegia, with auditory stimulation and attentional redirection identified as possible mechanisms of action (Zheng et al., 2023; Tegeler et al., 2025). In addition, experimental studies on slow-tempo music have shown that such music can enhance attentional orienting efficiency, lower heart rate, and increase subjective pleasure and relaxation (De Francesco et al., 2025; Husselman et al., 2026). Further support comes from a systematic review and meta-analysis on perioperative anxiety, which found that BB effectively reduced anxiety even in highly stressful situations, suggesting that their effects may be relatively stable across different contexts (Xiong et al., 2025). However, BB Chinese instrumental music may not produce significant anxiety-reducing effects among healthy undergraduate students, whereas anxiety relief has been observed among students with higher baseline trait anxiety (Pang et al., 2024). This suggests that the effects of BB music may be more likely to emerge among individuals with higher initial levels of anxiety or stress. Therefore, this finding provides an important rationale for focusing the present study on discipline inspection and supervision cadres as a high-stress occupational group.

Self-efficacy is commonly defined as individuals’ beliefs in their ability to successfully perform specific behaviors and achieve desired outcomes (Bandura, 1997). Existing studies have shown that higher self-efficacy and work engagement tend to use more positive emotion regulation to cope with stress (Schwarzer and Warner, 2012). Higher self-efficacy may make individuals more likely to focus their attention on music listening, thereby achieving greater immersion and relaxation (Zarza-Alzugaray et al., 2020; Zelenak, 2024). However, research remains limited on whether self-efficacy can further enhance its downstream effects on stress relief by improving music-listening experience.

In summary, three main gaps remain in the existing literature. First, previous studies have shown that music listening and BB music may be associated with anxiety relief, emotional improvement, and relaxation experiences; however, relatively few studies have focused on high-stress occupational groups in the public sector. Second, although self-efficacy and work engagement are considered important resources for coping with occupational stress, their relationships with psychological stress in the context of music listening have not been sufficiently examined. Third, it remains unclear whether music listening experience mediates the relationships among self-efficacy/work engagement, and psychological stress. Therefore, this study focuses on discipline inspection and supervision cadres as a high-stress occupational group, examines their subjective experiences following BB music listening, and further analyzes the relationships among self-efficacy, work engagement, music listening experience, and psychological stress. Specifically, this study aims to answer the following three research questions:

*RQ1*: After controlling for self-efficacy/work engagement, can music listening experience significantly predict psychological stress levels following a single exposure to binaural beat music?

*RQ2*: Does music listening experience mediate the relationship between self-efficacy/work engagement and psychological stress levels?

*RQ3*: What subjective experiences do discipline inspection and supervision cadres report after listening to binaural beat music?

## 3 Methods

### 3.1 Participants

This study employed a purposive sampling strategy to recruit in-service discipline inspection and supervision cadres from a region of Sichuan Province, China. An *a priori* power analysis was conducted using G*Power 3.1 (Faul et al., 2009) to determine the minimum sample size required for the hierarchical multiple regression (Research Question 1) and mediation analysis (Research Question 2). For the hierarchical regression testing the incremental contribution of music listening experience (one tested predictor) after controlling for self-efficacy (total of two predictors), a medium effect size of *f^2^* = 0.15 (Cohen, 1988) was assumed, with *α* = 0.05 and power (1–*β*) = 0.80. The analysis indicated that a minimum sample size of 55 participants was required. For the simple mediation model (self-efficacy → music listening experience → psychological distress), power estimates for the individual paths (a and b) also indicated a minimum of 55 participants for medium effects. Drawing on simulation-based recommendations for detecting indirect effects via percentile bootstrap (Fritz and MacKinnon, 2007), a sample size of at least 100–150 was considered adequate for medium-sized mediation effects. Considering potential incomplete responses and the need for robust estimation, the target sample size was set at 150–180. The final valid sample of *N* = 171 exceeded these requirements, ensuring adequate statistical power for both the hierarchical regression and mediation analyses.

The researchers distributed electronic questionnaires through internal channels of the discipline inspection and supervision system using the Wenjuanxing platform. This study was approved by the Human Research Ethics Committee of Universiti Sains Malaysia (USM/JEPeM/22120811). All participants provided electronic informed consent before completing the questionnaire. A total of 219 questionnaires were collected. To ensure data quality, all responses were screened according to predefined exclusion criteria: (1) an unusually short completion time of less than 30 s and (2) patterned responses, such as repeatedly selecting the same response option across consecutive items. After screening, 48 invalid questionnaires were excluded. The final sample included 171 valid questionnaires, corresponding to an effective response rate of 78.1%.

Participants were eligible if they were currently employed as discipline inspection and supervision cadres, were aged 18 years or older, and were able to independently complete the online questionnaire and music-listening procedure. Participants were excluded if they failed to complete the questionnaire, provided invalid responses (e.g., extremely short completion time or patterned responses).

### 3.2 Research procedure

This study employed a cross-sectional survey design and was conducted online, with data collected in April 2026. The electronic questionnaire was distributed through internal channels of the discipline inspection and supervision system to cadres in a region of Sichuan Province, China. Participants were provided with standardized written instructions and were advised to listen to the music in a quiet, distraction-free environment using their own headphones. Although the study was unable to control or record each participant’s ambient temperature or specific time of listening, clear instructions were provided to minimize external distractions as much as possible. After completing the music-listening session, participants proceeded to answer the remaining questionnaire items. The average time required to complete the questionnaire was approximately 3–5 min.

Following the quantitative survey, six participants were purposively selected for one-on-one semi-structured interviews based on the typicality of their responses, such as high reported stress levels or positive music-listening experiences, as well as their willingness to be contacted again. The interviews aimed to obtain a deeper understanding of participants’ subjective experiences and feelings during and after music listening. All interviews were conducted via online voice calls, audio-recorded with participants’ consent, and transcribed verbatim for subsequent qualitative analysis.

### 3.3 Research instruments

#### 3.3.1 Binaural beat music track

A 15-min BB music track was embedded in the questionnaire interface so that participants could listen to it before filling out the questionnaire. The 15-min listening duration was determined based on the length of the selected music segment, allowing participants to complete a relatively intact listening experience. The track was selected and edited from the album *Quantum Theta Waves: Binaural beat music for Meditation, Deep Relaxation and Healing* by David Gordon and Steve Gordon, released in 2020. The album integrates theta-frequency BB (primarily in the 4–8 Hz range, with specific tracks utilizing frequencies such as 6.8 Hz and 6.1 Hz) into soothing instrumental arrangements featuring ambient and light instrumental elements, without vocals (Gordon and Gordon, 2020).

#### 3.3.2 Psychological stress composite

This part measured participants’ emotional state and trait anxiety levels. Items were adapted from Zung (1971) Self Rating Anxiety Scale (SAS), with Cronbach’s *α* coefficients generally ranging from 0.70 to 0.90. The STAI includes separate measures of state and trait anxiety, both of which have shown high reliability, with Cronbach’s α coefficients typically exceeding 0.85 across different populations (Spielberger et al., 1983). Based on the actual questionnaire data, 19 valid items were included, rated on a 5 point Likert scale. Representative items included: “I feel calm,” “I feel secure,” “I am tense,” “Unimportant thoughts keep bothering me and interrupt me,” “When I think about my current work and tasks, I become tense.” Some items were reverse scored.

#### 3.3.3 Self-efficacy/work engagement composite

This part measured participants’ focus, sense of control, and self-efficacy at work. Items were adapted from Schwarzer and Jerusalem's (1995) General Self Efficacy Scale (GSES), with Cronbach’s α coefficients ranging from 0.76 to 0.90. Work engagement was assessed using three items adapted from the Utrecht Work Engagement Scale (UWES) (Schaufeli et al., 2002), as applied by Salanova et al. (2005). In the study by Salanova et al. (2005), the Cronbach’s α coefficients were 0.74 for vigor, 0.70 for dedication, and 0.77 for absorption. Based on the actual questionnaire data, 9 valid items were included, rated on a 5 point Likert scale (1 = strongly disagree, 5 = strongly agree). Representative items included: “I feel capable of meeting the high demands of my current situation,” “I am completely focused on the task at hand,” “I feel fully in control of what I am doing.”

#### 3.3.4 Self-reported music listening experiences

This section was developed with reference to the item design of the “stress regulation” and “cognitive regulation” factors of the Adaptive Functions of Music Listening Scale (AFML) developed by Groarke and Hogan (2018), and drew on the measurement logic of the “calmness” and “tension” dimensions of the Music Mood-Regulation Scale (MMRS) developed by Hewston et al. (2008). Based on the actual questionnaire data, four core outcome items were included and rated on a five-point Likert scale ranging from 1 (strongly disagree) to 5 (strongly agree). The items included: “While listening, I felt physically and mentally relaxed,” “My attention was naturally attracted by the music, and I was not easily distracted,” “The music helped me temporarily put aside work-related stress and worries,” and “After listening, I felt more emotionally stable than before.”

The internal consistency and structural validity of the four subjective music listening experience items were examined. The results showed that the Cronbach’s *α* coefficient was 0.79, with a Bootstrap 95% confidence interval of 0.729–0.835. The standardized Cronbach’s α coefficient was 0.791, and McDonald’s *ω* coefficient was 0.793, indicating good internal consistency. The corrected item–total correlations ranged from 0.579 to 0.633. The Cronbach’s α coefficient decreased when any item was deleted, indicating that each item contributed positively to the internal consistency of the measure.

The structural validity analysis showed that the KMO value was 0.57, and Bartlett’s test of sphericity was significant, *χ*^2^(6) = 422.88, *p* < 0.001. Parallel analysis suggested the extraction of two factors, which together explained 91.46% of the total variance. The oblique rotation results indicated that Items 30 and 31 mainly reflected participants’ immediate state during music listening, whereas Items 32 and 33 mainly reflected perceived stress and emotion regulation outcomes after music listening. The two-factor confirmatory factor model showed an acceptable overall fit, *χ*^2^(1) = 1.72, *p* = 0.190, CFI = 0.998, TLI = 0.990, RMSEA = 0.065, and SRMR = 0.009.

Regarding content validity, the item design was informed by existing music listening measurement instruments. Two experts with backgrounds in music education psychology were invited to review the items. Based on the experts’ feedback, the authors made revisions to the wording of items. Based on the current results, this measure was regarded as an exploratory instrument with preliminary reliability and validity evidence.

### 3.4 Interview guide

To gain an in depth understanding of participants’ subjective experiences and changes in feelings during the BB music listening, a semi structured interview guide was developed. The interviews covered five main topics:

(1) Emotional change trajectory: How did participants’ emotional states change during the music listening process?
(2) Key moments of stress relief: Did participants feel a clear reduction in irritation or anxiety at any particular moment? What characterized that moment?
(3) Subjective differences from ordinary music: Compared to ordinary music they usually listen to, what different experiences did BB music bring?
(4) Overall impression of digital intervention: What were their views on this mobile device based digital psychological intervention? How did they feel about its convenience and privacy protection?
(5) Personal and environmental factors affecting intervention outcomes: Did factors such as work busyness, personal preference for music, and listening environment influence their experience?

Six participants were interviewed. They were purposefully selected from those who completed the questionnaire and agreed to follow up contact. Each interview lasted about 20–30 min and was recorded with consent.

### 3.5 Data analysis

This study used a mixed data analysis method with quantitative analysis as the primary approach and qualitative analysis as supplementary.

(1) Quantitative data analysis. Quantitative data were analyzed using SPSS 26.0 and PROCESS macro (Hayes, 2018). First, descriptive statistics were calculated to examine the reliability of the scales. Second, hierarchical multiple regression analysis was conducted to address Research Question 1. self-efficacy and work engagement were entered in Block 1, the music listening experience added in the Block 2 to test its incremental predictive validity on psychological distress. Third, mediation analysis (Model 4 in PROCESS) with 5,000 bootstrap resamples was performed to address Research Question 2, examining whether music listening experience mediated the relationship between self-efficacy/work engagement and psychological stress. Standardized coefficients (*β*), *p*-values, and 95% confidence intervals for the indirect effect were reported. All statistical tests were two-tailed with a significance level of *α* = 0.05.

(2) Qualitative data analysis. Following Braun and Clarke’s (2006) thematic analysis framework, the interview transcripts were coded and organized into initial codes, sub-themes, and overarching themes. First, the researchers repeatedly read the interview transcripts to identify and mark meaningful information segments, thereby generating initial codes. Second, these initial codes were categorized and integrated according to their conceptual similarities and semantic relationships, leading to the development of sub-themes, namely second-level codes. Finally, based on these sub-themes, broader core themes were further refined and extracted to reflect the characteristics of the participants’ experiences, forming third-level codes. To enhance the credibility and consistency of the coding process, the research team discussed and reviewed the coding results collectively, compared interpretations of key excerpts. In addition, the data were examined through multiple rounds of coding and theme review. No substantially new themes emerged during the later stages of analysis; therefore, the data were considered to have reached thematic saturation. The analysis ultimately identified two core themes, each consisting of several sub-themes and supported by corresponding initial codes.

## 4 Results

### 4.1 Descriptive statistical analysis

The final analytic sample included 171 participants, of whom 66 were males (38.6%) and 105 were females (61.4%). Participants represented a wide age range, with the largest proportion aged 18–25 years (*n* = 63, 36.8%), followed by those aged 26–30 years (*n* = 35, 20.5%) and 31–40 years (*n* = 30, 17.5%). The remaining participants were aged 41–50 years (*n* = 18, 10.5%), 51–60 years (*n* = 12, 7.0%), and over 60 years (*n* = 13, 7.6%). Descriptive statistics were calculated to summarize participants’ psychological stress-related responses, self-efficacy/work engagement, and subjective music listening experiences. Because relevant stress-related items had been reverse-scored, higher scores indicated stronger endorsement of stress- or anxiety-related responses. Relatively high mean scores were observed for reduced calmness and composure (Q10, *M* = 3.13, *SD* = 1.39), nervousness (Q3, *M* = 3.00, *SD* = 1.32), work-related tension (Q20, *M* = 2.99, *SD* = 1.40), and intrusive thoughts (Q17, *M* = 2.97, *SD* = 1.33). These results suggest that participants reported a moderate level of psychological stress-related experiences. At the same time, participants also reported some coping and work-related resources, including perceived ability to meet current demands (Q21, *M* = 3.10, *SD* = 1.34) and task absorption (Q25, *M* = 3.03, *SD* = 1.39). Following the single session of BB music listening, participants reported relatively positive subjective music-listening experiences, including relaxation (Q30, *M* = 3.88, *SD* = 0.73), attentional engagement (Q31, *M* = 3.87, *SD* = 0.71), temporary stress relief (Q32, *M* = 3.99, *SD* = 0.70), and improved emotional stability (Q33, *M* = 3.98, *SD* = 0.73).

### 4.2 Hierarchical regression analysis predicting psychological stress

A two-step hierarchical multiple regression analysis was conducted to examine whether music listening experience explained additional variance in psychological stress following a single session of BB music, beyond self-efficacy/work engagement. In Step 1, the self-efficacy/work engagement composite was entered as the predictor. This model was statistically significant, *R^2^* = 0.026. *F* (1, 169) = 4.44, *p* = 0.037. Self-efficacy/work engagement negatively predicted psychological stress (*B* = −0.183, *SE* = 0.087, *β* = −0.160*, t* = −2.11, *p* = 0.037) (see Table 1).

**Table 1:** Hierarchical multiple regression predicting psychological stress.

Variable	*B*	*SE B*	*β*	*t*	*p*
Step 1					
Self-efficacy and work engagement	−0.183	0.087	−0.160	−2.11	0.037
Model summary	*R*^2^ = 0.026, *F* (1, 169) = 4.48, *p* = 0.036				
Step 2					
Self-efficacy and work engagement	−0.165	0.081	−0.144	−2.03	0.044
Music listening experience	−0.340	0.067	−0.361	−5.08	< 0.001
Model summary	*R*^2^ = 0.155, Δ*R*^2^ = 0.130, *F* (2, 168) = 15.45, *p* < 0.001				

**Figure 1:**
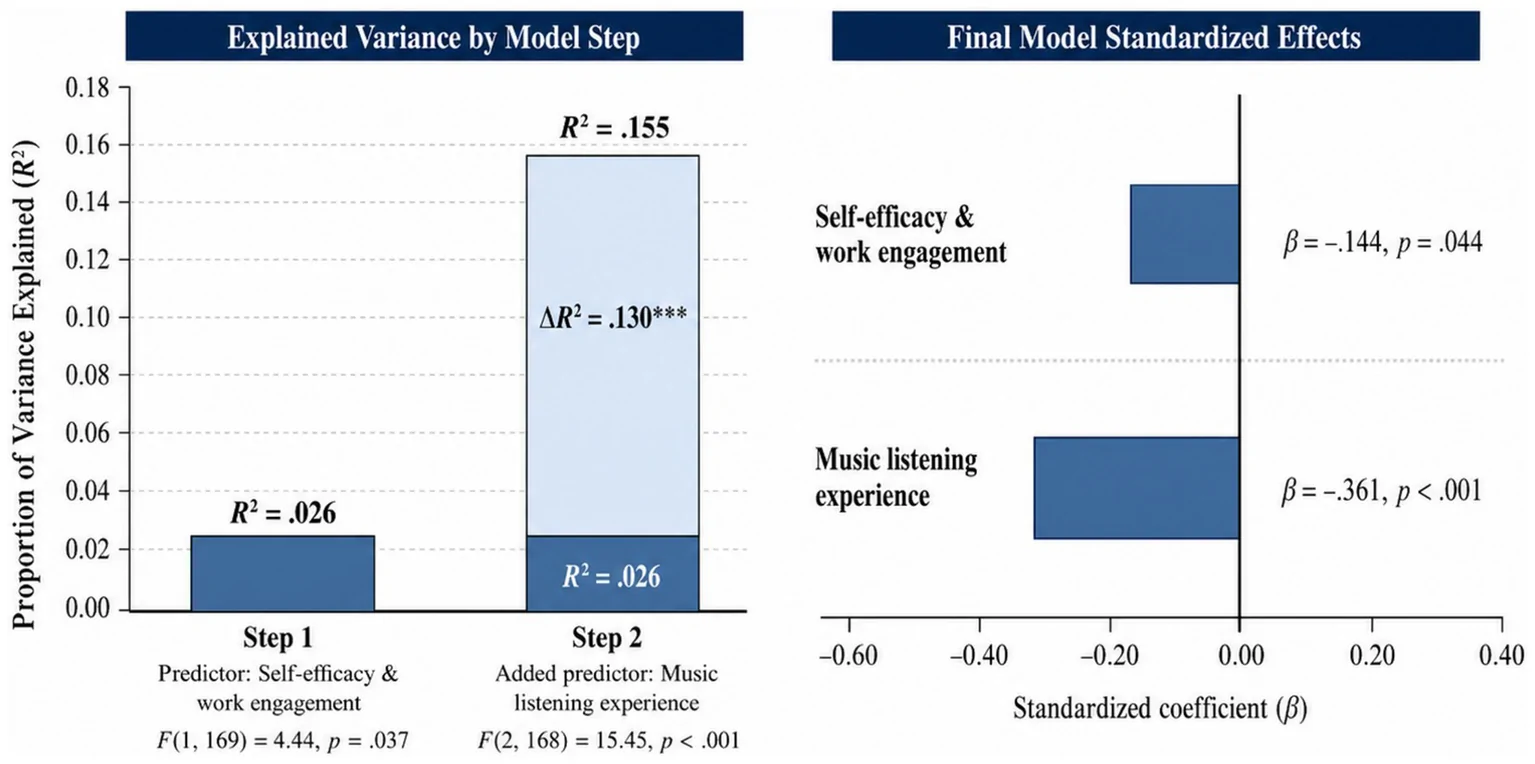
Incremental contribution of music listening experience to psychological stress.

In Step 2, music listening experience was added to the model. The addition of music listening experience significantly increased the proportion of variance explained, Δ*R^2^* = 0.130, *F* (1, 168) = 26.02, *p* < 0.001. The full model accounted for 15.5% of the variance in psychological stress, *R*^2^ = 0.155, *F* (2,168) = 15.45, *p* < 0.001. In the final model, both self-efficacy/work engagement (*β* = −0.165, SE = 0.081, *β* = −0.144, *t* = −2.03, *p* = 0.044) and music listening experience (*β* = −0.340, *SE* = 0.067, *β* = −0.361, *t* = −5.08, *p* < 0.001) emerged as significant negative predictors of psychological stress. As shown in Figure 1, the addition of music listening experience significantly increased the explained variance.

### 4.3 Mediation analysis

A mediation analysis was conducted to examine whether subjective music listening experience mediated the relationship between self-efficacy/work engagement and psychological stress following a single session of BB music. The results are presented in Table 2.

**Table 2:** Mediation analysis results: music listening experience as mediator.

Path/effect	Coefficient (B)	SE	*p*	Standardized β	95% bootstrap CI/note
a: X → M (self-efficacy/work engagement → music listening experience)	0.053	0.093	0.572	0.053	–
b: M → Y (music listening experience → psychological stress|X)	−0.340	0.067	<0.001	−0.361	–
c′: direct effect (X → Y|M)	−0.165	0.081	0.044	−0.144	–
ab: indirect effect	−0.018	–	–	–	[−0.090, 0.046]
c: total effect (X → Y)	−0.183	0.087	0.037	−0.160	–

The path from self-efficacy/work engagement to music listening experience (path *a*) was not statistically significant (*B* = 0.053, *p* = 0.572). By contrast, the path from music listening experience to psychological stress (path *b*), controlling for self-efficacy/work engagement, was significant and negative (*B* = −0.340, *p* < 0.001). The direct effect of self-efficacy/work engagement on psychological stress (path *c*′) remained significant after controlling for music listening experience (*B* = −0.165, *p* = 0.044). The total effect of self-efficacy/work engagement on psychological stress (path *c*) was also significant (*B* = −0.183, *p* = 0.037).

**Figure 2:**
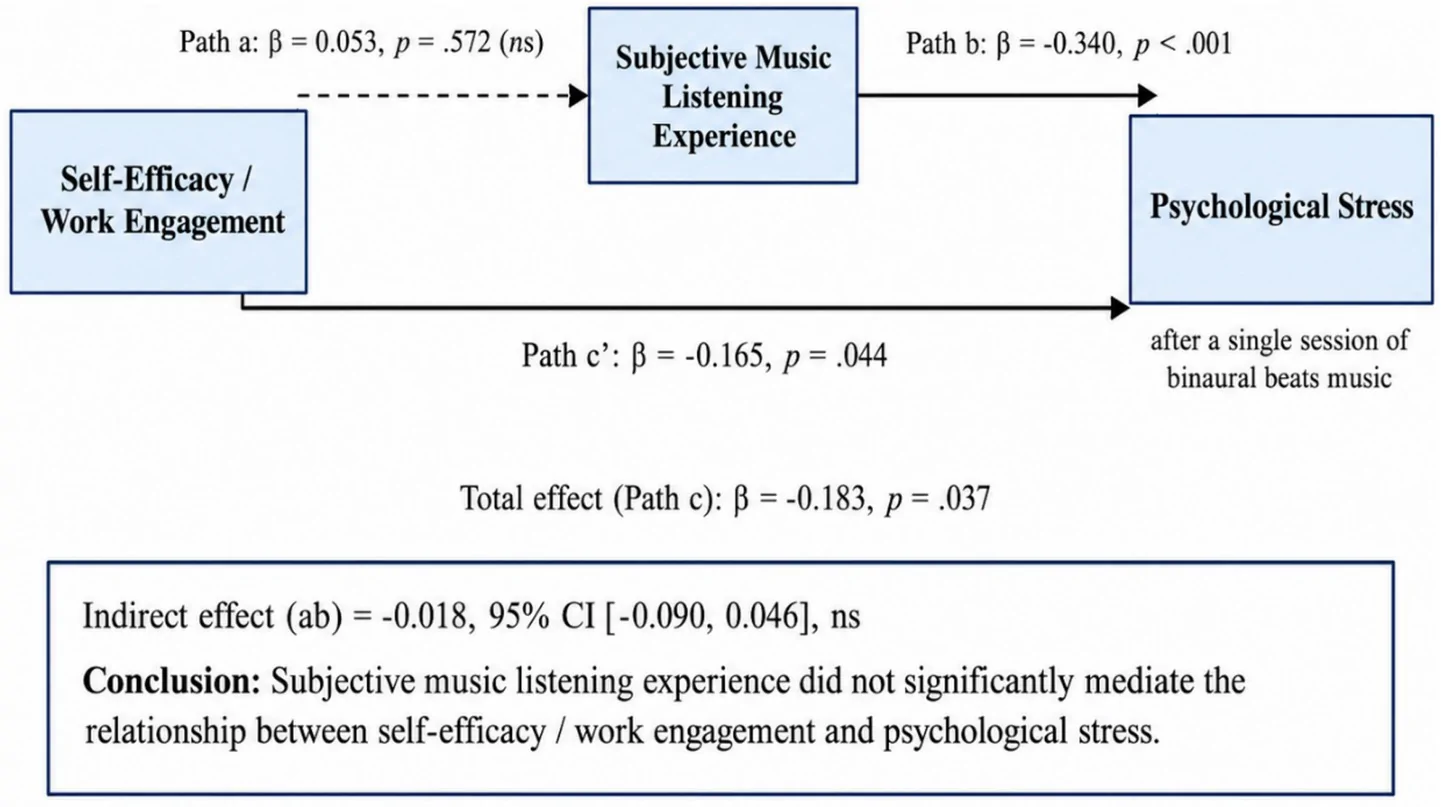
Mediation model of psychological stress following a single session of binaural beat music.

However, the indirect effect through music listening experience was small and statistically non-significant [*ab* = −0.018, 95% CI (−0.090, 0.046)], as the bootstrap confidence interval included zero (see Figure 2).

These results indicate that although both self-efficacy/work engagement and music listening experience were significantly associated with lower psychological stress, music listening experience did not significantly mediate the relationship between self-efficacy/work engagement and psychological stress.

### 4.4 Qualitative analysis results

The thematic analysis identified two overarching themes characterizing participants’ experiences of BB music listening. The first theme was Emotional Regulation and Positive Affective Activation, and the second theme was Toward Personalized and Sustainable Relaxation Support.

#### 4.4.1 Theme 1: emotional regulation and positive affective activation

Participants consistently described a positive shift in emotional states following the BB music listening session. This transformation was generally characterized by movement from heightened tension and anxiety toward greater calmness. Two interrelated patterns were identified.

##### 4.4.1.1 Sub-theme 1: transition from anxiety to calmness and relaxation

Many participants reported a gradual release of physical and mental tension during the session.

“At the beginning my mind was chaotic, thinking about all the unfinished files on my desk… Around the middle, I suddenly felt like I was being pulled out of those thoughts, and a sense of calmness gradually emerged as my mood settled considerably.” (T20260401).

“My heart felt like it was being clawed by a cat—I could not sit still. Later, when the music reached a particularly slow section, it was like a hand gently stroking from the top of my head downward, bringing a sense of relaxation as the anxiety was drawn away inch by inch.” (C20260405).

These accounts indicate that the music facilitated both cognitive disengagement from immediate stressors and somatic relaxation.

##### 4.4.1.2 Sub-theme 2: arousal of positive emotions

Beyond the reduction of negative affect, several participants reported the emergence of positive emotional states.

“After listening, I wasn’t just less irritable—as if I had been lifted out of the muddle I was in before. Things felt brighter inside.” (C20260405).

“When the final note faded, I noticed the corners of my mouth were actually upturned. It had been a long time since I felt this light.” (Z20260404).

These accounts suggest that the listening not only alleviated distress but also facilitated positive affective activation.

#### 4.4.2 Theme 2: toward personalized and sustainable relaxation support

Participants expressed both clear expectations and concerns about the digital BB music. While they valued its potential for personalization and convenience, they also raised questions about the sustainability of its effects. These views clustered into three sub-themes.

##### 4.4.2.1 Sub-theme 1: expectations for personalized recommendations

“If it could sense whether I’m anxious or low that day and automatically match different music, that would be really thoughtful. Sometimes I need relaxation, sometimes I need something uplifting.” (Z20260404).

“The piece I listened to today worked well, but tomorrow my mood might be different and I may want something else. If it had a simple mood-selection interface so I could choose according to my state that day, I would be more willing to use it long-term.” (L20260408).

“I prefer pure instrumental music. Ideally it would learn my preferences and get better at knowing me over time. Otherwise, if it’s always the same, I’ll definitely get bored after a while.” (T20260401).

##### 4.4.2.2 Sub-theme 2: concerns about the durability of effects

“While listening, my whole body did loosen up, but as soon as I turned it off and returned to reality, the stress came flooding back. I worry this is just temporary escape and the effect will not last long.” (M20260408).

“If it could have a longer-lasting effect, or if I could somehow call that state back whenever needed, it would be better.” (C20260405).

##### 4.4.2.3 Sub-theme 3: accessible relaxation support

“The biggest feeling was ‘slowing down’… My heartbeat wasn’t so urgent, and the sense of being chased temporarily disappeared. And I can use it just with my phone—that’s very convenient for me.” (M20260408).

“We often have to travel or conduct interviews and cannot have fixed times or places for relaxation. Having such a portable tool reduces the psychological burden a lot.” (T20260401).

Overall, the qualitative findings suggest that BB music listening was experienced as a short-term means of reducing anxiety, promoting relaxation, and supporting emotional regulation. However, participants’ accounts also indicate that future applications should move beyond one-time listening experiences by incorporating personalized recommendation functions, supporting repeated use, and enhancing the sustainability of emotional benefits.

## 5 Discussion

Hierarchical regression analysis showed that self-efficacy/work engagement was significantly and negatively associated with psychological stress, indicating that participants with higher levels of self-efficacy/work engagement tended to report fewer stress-related experiences. This finding is consistent with previous research suggesting that self-efficacy, as an important personal psychological resource, can help individuals cope with occupational stress and emotional challenges (Schwarzer and Warner, 2012). More importantly, after controlling for self-efficacy/work engagement, music listening experience remained a significant negative predictor of psychological stress (*β* = −0.361, *p* < 0.001). The inclusion of music listening experience also increased the explained variance in psychological stress by 13.0% (Δ*R*^2^ = 0.130). This finding supports previous evidence linking music-based interventions with reductions in stress, anxiety, and emotional distress (de Witte et al., 2022; Nyarubeli et al., 2025). Its significance lies in showing that music listening experience provided additional explanatory value beyond self-efficacy and work engagement. This relationship may reflect the distinctive functions of music in redirecting attention, regulating emotion, and promoting relaxation, thereby highlighting its potential therapeutic value.

The mediation analysis showed that music listening experience did not significantly mediate the relationship between self-efficacy/work engagement and psychological stress. Although previous research has suggested that higher self-efficacy may help individuals sustain attention and participate more actively in musical activities (Zarza-Alzugaray et al., 2020). Self-efficacy/work engagement did not significantly predict music listening experience in the present study (path *a*, *p* > 0.05). It is important to note that both self-efficacy/work engagement and music listening experience were significantly associated with lower psychological stress. However, the data did not support the proposed pathway in which self-efficacy/work engagement was associated with psychological stress through enhanced music listening experience. One possible explanation is that self-efficacy/work engagement and music listening experience may operate through different psychological pathways. Self-efficacy/work engagement primarily reflects individuals’ beliefs about their own capabilities and their work-related psychological state, which may support stress regulation by fostering a more positive and confident approach to occupational demands (Schwarzer and Warner, 2012). By contrast, music listening may alleviate stress through melody, rhythm, emotional resonance, attentional redirection, and relaxation (Elnazer, 2026; Raittila et al., 2025; Zheng et al., 2023). Accordingly, self-efficacy/work engagement and music listening experience may be associated with psychological stress through relatively independent pathways rather than through a sequential mediating process. This finding suggests that music-based support programs for high-stress occupational groups should address these two pathways separately: enhancing self-efficacy and work engagement while also optimizing the listening experience according to individuals’ immediate emotional states and musical preferences. These approaches may provide complementary forms of support for stress management.

The qualitative findings further complemented and extended the quantitative results. Participants commonly described BB music listening as a process of moving from tension and anxiety toward greater calmness and relaxation. This finding is consistent with de Witte et al. (2020), who identified attentional redirection and emotion regulation as important pathways through which music interventions may be associated with stress relief. Participants’ evaluations of the delivery format also indicated that convenience may be an important reason for the potential applicability of BB music among high-stress occupational groups. Future applications could provide personalized options based on users’ immediate emotional states, musical preferences, and listening contexts. Repeated listening and integration with other stress-management strategies should also be examined to determine whether the reported benefits can be sustained over time.

This study has several limitations. First, because it employed a cross-sectional design and examined only a single session of BB music listening, the findings cannot be interpreted as causal evidence that BB music directly reduces psychological stress. Future studies should adopt experimental designs to determine whether repeated listening can produce sustained improvements in psychological stress among high-pressure occupational groups. Second, self-efficacy and work engagement were analyzed as a composite predictor; therefore, the independent contribution of each construct should be interpreted with caution. Third, the study relied primarily on self-report data. Future research should incorporate objective measures such as Electroencephalography (EEG), heart rate, heart rate variability, electrodermal activity, or cortisol. Fourth, the music listening experience scale received preliminary expert review and showed initial evidence of structural validity, it remains an exploratory instrument and requires further validation across independent samples. Beyond these limitations, several aspects of the online listening procedure may also have affected the internal validity of the findings. Although participants were instructed to listen in a quiet environment without engaging in other activities, we did not record ambient temperature or the precise time of day. These environmental and temporal factors may have influenced participants’ psychological responses. Future research conducted in controlled laboratory settings with standardized temperature and timing would help strengthen internal validity.

## 6 Conclusion

In summary, this study examined BB music listening in the real-world occupational context of a high-stress public-sector group. By integrating hierarchical regression, mediation analysis, and qualitative analysis, the study provides preliminary evidence on the relationships among self-efficacy, work engagement, subjective music listening experience, and psychological stress. A key contribution is the finding that positive music listening experience explained additional variance in psychological stress beyond self-efficacy and work engagement. The results further suggest that self-efficacy/work engagement and music listening experience may be associated with psychological stress through relatively independent pathways, rather than through a mediating process. These findings extend research on BB music to high-stress occupational populations and provide an initial basis for developing convenient, low-cost forms of psychological support.

## Data Availability

The original contributions presented in the study are included in the article/supplementary material, further inquiries can be directed to the corresponding authors.
